# Beta Toxins Isolated from the Scorpion *Centruroides hirsutipalpus* (Scorpiones; Buthidae) Affect the Function of Sodium Channels of Mammals

**DOI:** 10.3390/toxins17120584

**Published:** 2025-12-06

**Authors:** Laura L. Valdez-Velazquez, Timoteo Olamendi-Portugal, Rita Restano-Cassulini, Lidia Riaño-Umbarila, Juana María Jiménez-Vargas, Fernando Zamudio, Hermenegildo Salazar-Monge, Baltazar Becerril, Lourival D. Possani

**Affiliations:** 1 Facultad de Ciencias Químicas y Facultad de Medicina, Universidad de Colima, Km 9 Carretera Colima, Coquimatlán s/n, Coquimatlán 28400, Mexico; lauravaldez@ucol.mx (L.L.V.-V.); jjimenez45@ucol.mx (J.M.J.-V.); 2 Facultad de Medicina, Universidad de Colima, Avenida Universidad 333, las Víboras, Colima 28040, Mexico; hsalazar@ucol.mx; 3 Departamento de Medicina Molecular y Bioprocesos, Instituto de Biotecnología, Universidad Nacional Autónoma de México, Cuernavaca 62210, Mexico; timoteo.olamendi@ibt.unam.mx (T.O.-P.); rita.restano@ibt.unam.mx (R.R.-C.); lidia.riano@ibt.unam.mx (L.R.-U.); fernando.zamudio@ibt.unam.mx (F.Z.); baltazar.becerril@ibt.unam.mx (B.B.); 4 Investigadora por México, Secretaría de Ciencia, Humanidades, Tecnología e Innovación Secihti, Av. Insurgentes Sur 1582, Col. Crédito Constructor, Bénito Juárez, Ciudad de México 03940, Mexico

**Keywords:** β-toxins, *Centruroides hirsutipalpus*, voltage-gated sodium channels, scorpion

## Abstract

Scorpion venom toxins are important peptides being studied for their clinical significance. These peptides act by binding to ion channels in the membrane of nerve cells, causing the symptoms associated with scorpion stings (scorpionism). They principally affect the function of voltage-gated sodium channels (Nav) and are valuable for studying ion channels. Scorpions from the Buthidae family contain toxins that affect sodium channels and have a high affinity for mammalian channels. In this study, two sodium toxins isolated from the venom of the scorpion *Centruroides hirsutipalpus*, a member of the Buthidae family, were identified as belonging to the beta-type subfamily. These toxins were purified from whole venom using molecular exclusion, cationic-exchange, and reverse-phase chromatography techniques. Their molecular masses were determined using mass spectrometry, while their amino acid sequences were obtained by Edman degradation. A comparative analysis revealed that the sequences are identical to ChiNaBet60 and ChiNaBet50 toxins (now named Chirp7 and Chirp9, respectively) previously identified in the venom gland transcriptomics from *C. hirsutipalpus*. Furthermore, toxicity studies showed that these toxins were lethal to mammals. Electrophysiological analysis revealed that these peptides act as sodium channel–modulating toxins. In addition, interaction assays with antibodies were performed to analyze the structural determinants governing the binding mechanism.

## 1. Introduction

Scorpion venom is a secretion from a gland where molecular machinery generates multiple protein substances and many active compounds of various chemical classes [1]. Some of these components target the nervous system, disrupting cellular communication by modifying or blocking signals between nerves and muscles, while others degrade molecules, causing cell and tissue breakdown [2,3]. In scorpion venom, the majority of the toxic components are peptides that interact with sodium and potassium channels, with the sodium toxins being more abundant in medically significant scorpion venoms [4,5,6,7]. When sodium toxins bind to the sodium channels, they alter their normal function and disrupt the propagation of electrical impulses along the nervous system and muscles [8]. These alterations can lead to neuronal hyperexcitability and the generation of symptoms such as intense pain, muscle contractions, paralysis, and even death in severe cases [5,9].

*Centruroides hirsutipalpus* is an endemic scorpion species found in the region of western Mexico named Minatitlán, Colima. This species is closely related morphologically and geographically to *Centruroides tecomanus*; both species are known as striped scorpions and are considered medically important [10]. *Centruroides hirsutipalpus* is categorized as one of the most dangerous species in Mexico, given that transcriptomic and proteomic analyses have revealed the presence of toxins that target sodium channels, which can lead to human fatalities [11]. Moreover, the soluble venom of *C. hirsutipalpus* has been evaluated in cells stably expressing human sodium channels from hNav1.1 to hNav1.7, showing activity on the hNav1.1, hNav1.2, and hNav1.6 subtypes. In these channels, venom triggers channel activation at more negative potentials and reduces the peak current like the beta scorpion toxins [12].

The study of sodium toxins from the scorpion *C. hirsutipalpus* is of great scientific and medical interest. Understanding the effect of these toxins can contribute to developing new drugs and improving antivenoms. Furthermore, research on these toxins also enhances our knowledge of scorpion biology and their corresponding venoms.

## 2. Results and Discussion

**Figure 1:**
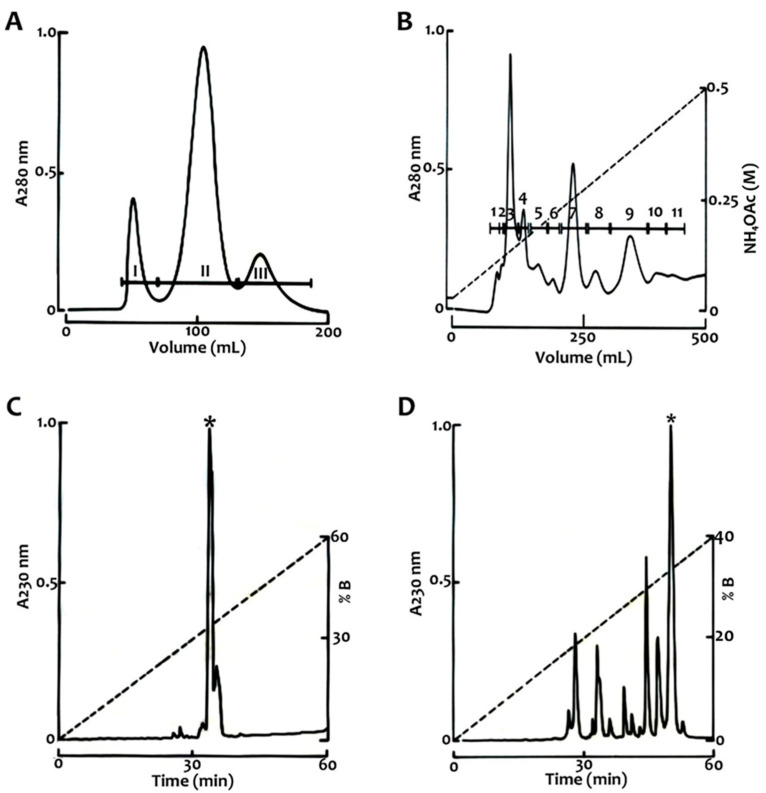
Purification of two toxins from the venom of *C. hirsutipalpus*. (**A**) shows the Sephadex G-50 separation profile of 96 mg of soluble venom, three fractions were observed: I, II and III. The toxic fraction II, containing 63% of the total protein, was further separated by CM-cellulose ion-exchange chromatography (**B**), yielding 11 subfractions. Components II-7 (**C**) and II-9 (**D**), each recovered at approximately 0.75 mg of protein, were obtained in pure form (asterisks) after purification by HPLC on a Vydac C18 reverse-phase column (4.6 × 250 mm), as described in Section 4.

In this report, we identified two toxin peptides in the venom of *C. hirsutipalpus* that affect sodium channels. To analyze these peptides, we separated the venom using a Sephadex G-50 column, which yielded three typical fractions similar to those found in other venoms of the Centruroides genus [13,14] (Figure 1A). Fraction II was subsequently purified through cationic-exchange chromatography, resulting in eleven distinct fractions (Figure 1B). The predominant peaks from the ion-exchange chromatogram were fraction II.3 (previously reported in Valdez-Velazquez, 2018) [12], fraction II.7 (Chirp7), and fraction II.9 (Chirp9). These fractions were previously identified in the transcriptome of *C. hirsutipalpus* [11]. Subsequently, these peptides underwent further purification by HPLC (Figure 1C,D).

In toxicological assays, the toxins Chirp7 (component II-7) and Chirp9 (component II-9) exhibited pronounced lethality in murine models. Following intraperitoneal administration, affected animals presented a consistent set of symptoms, including hyperexcitability, tremors, piloerection, hypersalivation, transient paralysis, dyspnea, motor incoordination, severe respiratory distress, and, ultimately, death approximately 20 min after injection.

Table 1 shows the toxicological profile of fractions obtained from cation-exchange chromatography. Fractions (FII.1–FII.9) were first separated by cation-exchange chromatography and subsequently analyzed by reverse-phase HPLC, from which the retention times (RT) were obtained. The table includes the fraction number, molecular mass, HPLC retention time, and the toxicological effects observed in mice. Notably, fractions FII.7 and FII.9 showed particularly lethal effects in mice.

**Figure 2:**
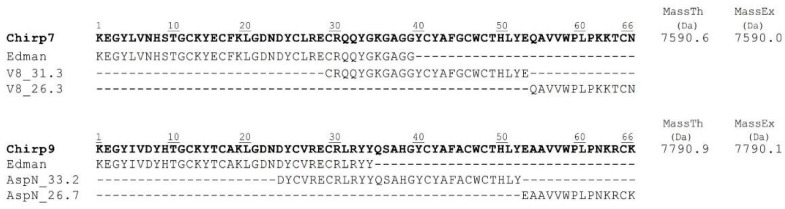
Chirp7 and Chirp9 peptide sequences were obtained after reduction, carboxymethylation, and digestion with V8 and Asp-N endopeptidases using the Edman degradation method. Their molecular mass was determined by mass spectrometry.

The mass and complete amino acid sequences of these subfractions were determined. For Chirp7 (UniProt C0HMC5), the first 39 amino acids in the N-terminal region were identified through Edman degradation. An overlapping segment at the C-terminal section, specifically residues 29 to 66, was identified following reduction, carboxymethylation, and digestion with endopeptidase V8. In the case of Chirp9 (UniProt C0HMC6), the first 34 amino acids at the N-terminal region were also identified directly by Edman degradation. An overlapping segment in the C-terminal section, from residues 23 to 66, was identified after reduction, carboxymethylation, and digestion with endopeptidase Asp N (Figure 2).

Both Chirp7 and Chirp9 peptides are composed of 66 amino acids and contain eight cysteine residues that form four disulfide bridges. These peptides were identified through transcriptomic analyses (ChiNaBet60 and ChiNaBet50) and are classified as beta-type sodium scorpion toxins (β-NaScTxs; Valdez-Velázquez et al., 2020) [11]. Notably, these toxins possess a C-terminal amidation, a post-translational modification known to enhance their biological activity [15]. Sodium toxins (NaScTxs) are divided into two main categories: alpha toxins (α-NaScTxs), which bind to site 3 of the sodium channel [8,16], and beta toxins (β-NaScTxs), which bind to site 4 [17,18,19]. Additionally, β-NaScTxs are categorized into insect-selective β-NaScTxs [20,21,22,23], mammalian-selective β-NaScTxs, and β-like NaScTxs [24]. Sodium toxin peptides have been extensively studied due to their clinical significance, their diversity, and the interest in understanding their pharmacological and electrophysiological actions.

**Figure 3:**
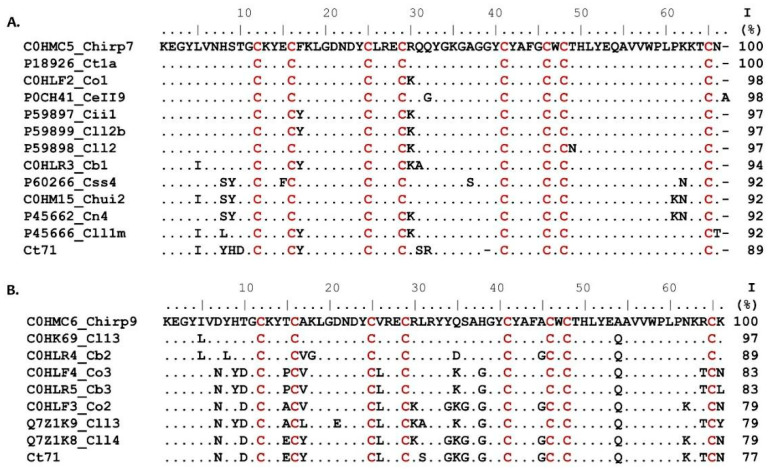
Sequence alignment of Chirp7 (**A**) and Chirp9 (**B**) toxins, both derived from the *C. hirsutipalpus* scorpion, alongside associated sodium toxins. Conserved cysteine residues are highlighted in red, and dots indicate identical residues. The peptides are aligned with sequences obtained from UniProt, followed by the toxin name. The percentage of identity (%ID) was calculated based on the mature peptide sequence. Sequence alignments were performed using BLASTp version 2.11.0 [26] via the NCBI BLAST web server (accessed on 14 June 2024).

When comparing the sequence of the new toxins β-NaScTxs from *C. hirsutipalpus* with sequences from different scorpions (Figure 3A) using BLASTp version 2.11.0, it is shown that the sequence of the toxin Chirp7 is 100% identical to the sodium toxin Ct1a (UniProt accession P18926) from the *C. tecomanus* scorpion. Ct1a is known for its effect on the hNav1.6 channel, altering channel activation by lowering the action potential and decreasing the current amplitude, typical traits of beta toxins [13]. β-NaScTxs toxins modify sodium currents by activating a voltage sensor-trapping mechanism [18] that results in the channel activation to more hyperpolarized potentials [25].

Although both scorpions (*C. hirsutipalpus* and *C. tecomanus*) are found in the same Pacific region of Mexico, they have habitats separated by approximately 90 km. *C. hirsutipalpus* is endemic to a forested and mountainous area, with an average altitude of 742 m above sea level. On the other hand, *C. tecomanus* inhabits coastal plains with an average altitude of 30 m above sea level. Venoms contain various toxins that can differ depending on the species, habitat, or environmental conditions [27]. Chirp7 constitutes 8.9% of the total venom in *C. hirsutipalpus*, with an experimental molecular weight of 7590 Da and a theoretical mass of 7590.6 Da. The abundance of Chirp7 in *C. hirsutipalpus* venom is significantly higher than that of Ct1a toxin, which makes up only 1.7% of *C. tecomanus* venom [13]. On average, *C. hirsutipalpus* scorpions produce 377 ± 81 μg of venom proteins, while *C. tecomanus* scorpions contain 334 ± 66 μg when they are milked by electrical stimulation. The higher abundance of Chirp7 in *C. hirsutipalpus* venom suggests that it may act faster in the envenomation process as compared to Ct1a toxin. In *C. tecomanus* venom, the most abundant toxin is Ct71, which constitutes 7.3% of the venom [28]. Notably, Ct71 shares an 89% sequence identity with Chirp7 (Figure 3A). Furthermore, *C. hirsutipalpus* venom likely contains additional toxins that synergistically contribute to envenomation symptoms, thereby increasing its overall toxicity. The Chirp7 sequence also shares high identity (ranging from 98% to 92%) with other peptides, including the Co1 of *C. ornatus* [29], CEII9 from *C. elegans* [30], Cii1 toxin from *C. infamatus* [31], ClI2, ClI2b, and ClI1m from *C. limpidus* [25], Cb1 from *C. baergi* [28], Css4 from *C. suffusus* [32], Chui2 from *C. huichol* [28], and Cn4 from *C. noxius* (Figure 3A) [33].

The Chirp9 peptide in the venom of *C. hirsutipalpus* has an abundance of 2.8%. It has an experimental mass of 7790.1 Da and a theoretical mass of 7790.9 Da. The sequence of Chirp9 (Figure 3B) shares 97% identity with the Cl13 toxin from the *C. limpidus* scorpion (UniProt C0HK69) and differs by only two amino acid substitutions: L5I and Q54A. These substitutions could modulate the toxic properties of the venom through structural alterations in the peptide that influence its interaction with target sites, possibly leading to increased potency or stability. The Cl13 toxin is found in *C. limpidus* venom with an abundance of 2.1% [28]. This venom has a median lethal dose (LD_50_) of 15 μg/20 g in CD1 mice. In contrast, the LD_50_ for *C. hirsutipalpus* venom is 12 μg/20 g [28]. The Chirp9 toxin is present in slightly higher abundance than Cl13 in *C. limpidus*. It is known that Cl13 is a beta toxin that modifies sodium currents of the channels Nav1.2, Nav1.4, Nav1.5, and Nav1.6 [34]. Furthermore, the sequence of Chirp9 shows 89–79% identity with other toxins, including Cb2 and Cb3 from *C. baergi* [28], Co2 and Co3 from *C. ornatus* [29], and Cll3 and Cll4 from *C. limpidus* [34].

**Figure 4:**
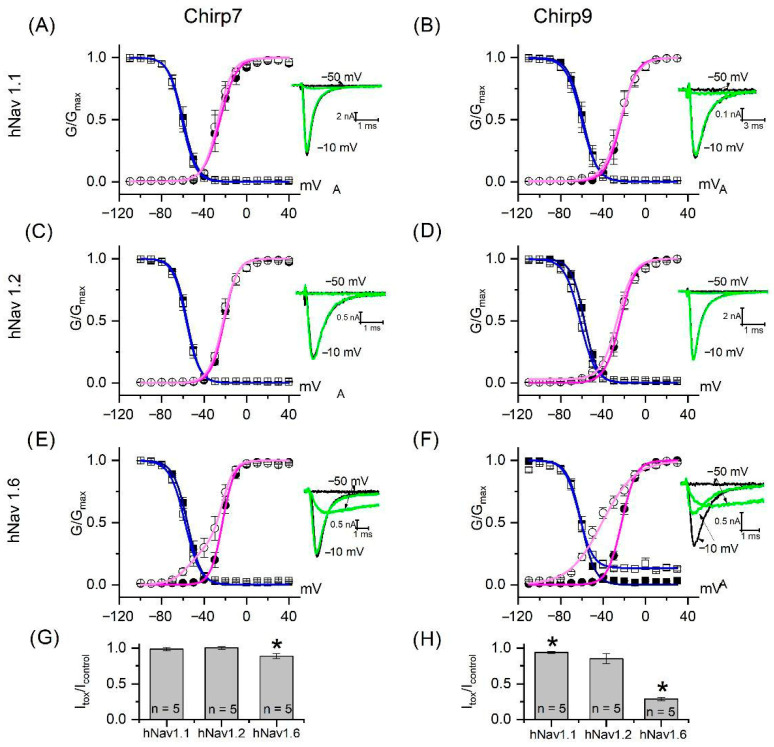
Effect of Chirp7 and Chirp9 on sodium channels hNav1.1, 1.2, and 1.6. Sodium currents were elicited by the stimulation protocol described in the Section 4 and depicted in Supplementary Figure S1. The right panels of (**A**–**F**) show the currents recorded at −50 mV (under activation potential) and −10 mV (full activation potential) in the control (black traces) and after toxin exposure (green traces). Currents are plotted against membrane potentials and representative activation (circles) and inactivation (squares) curves for hNav1.1 (**A**,**B**), hNav1.2 (**C**,**D**), and hNav1.6 (**E**,**F**) channels before (black symbols) and after (open symbols) exposure to 200 nM Chirp7 (**A**,**C**,**E**) or Chirp9 (**B**,**D**,**F**). Magenta and blue traces are the best fit for the data sets. Fitting parameters are reported in Table S1. Panels (**G**,**H**) summarize the residual peak current calculated after application of Chirp7 and Chirp9, respectively. Data are presented as mean ± SE; and an asterisk (*) indicates a statistically significant difference at the 0.05 level as determined by a paired sample *t*-Test.

In the study of Valdez-Velázquez et al. (2018) [12], the soluble venom of *C. hirsutipalpus* was evaluated at a concentration of 20 μg/mL against seven human voltage-gated sodium channel subtypes (hNav1.1–1.7) and two potassium channel subtypes (hKv1.1 and hKv11.1). The venom elicited a β-type modulation of sodium channel gating, characterized by a leftward shift in the voltage dependence of activation and a progressive reduction in peak current amplitude, in hNav 1.1, hNav 1.2, and hNav 1.6. This modulatory profile is consistent with the canonical mechanism of scorpion β-toxins that promote channel activation at more negative potential and reduction in the peak current, early described as “voltage sensor trapping” [17,35,36]. Based on these observations, the isolated peptides Chirp7 and Chirp9 were specifically assayed on hNav1.1, hNav1.2, and hNav1.6 channels (Figure 4). If complete venom has not altered the currents of the hNav 1.3, hNav 1.4, hNav 1.5, hNav 1.6 and 1.7 channels, there is no reason to think that isolated poisons could interfere with their proper functioning.

Both peptides, at 200 nM, induced a leftward shift in the activation curves of hNav1.6, where Chirp7 and Chirp9 also reduced the peak current (Figure 4E–H; Table S1). In hNav1.6, Chirp9 also slows down the inactivation kinetic by promoting a residual current that can be observed in the activation curve at potentials greater than −30 mV, whereas in control conditions channels are completely inactivated (Figure 4F).

The ability of scorpion beta toxins to slow down the kinetics of sodium channel inactivation is not new and has also been reported for Cl13, a toxin that differs from Chirp9 by only two amino acids. In contrast, Chirp7 and Chirp9 were unable to modify the kinetics of hNav 1.1, while Chirp9 shifted inactivation to more negative potential in hNav1.2 channels. These results confirm that Chirp7 and Chirp9 are responsible, at least in part, for the effect previously observed in *C. hirsutipalpus* venom.

Since *C. hirsutipalpus* soluble venom also produces a beta effect in hNav 1.1 and hNav 1.2 currents [12], it is reasonable to assume that the venom of *C. hirsutipalpus* contains other sodium toxins that participate in the poisoning process.

**Figure 5:**
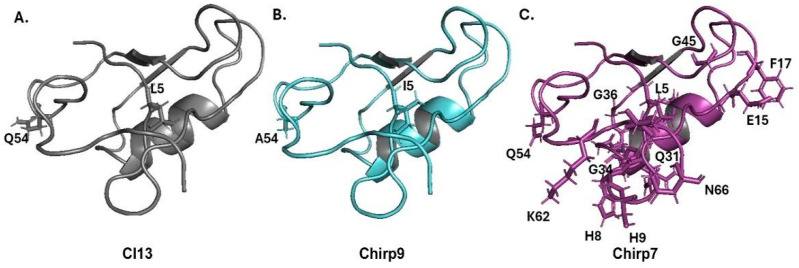
Structural representation of the Cl13 (**A**), Chirp9 (**B**), and Chirp7 (**C**). Each toxin is represented on the carton and has different amino acid residues compared to the Chirp9 toxin, which is shown in sticks.

As already mentioned, Chirp9 toxin exhibits only two amino acid differences compared to the Cl13: I5L and A54Q. Despite these seemingly minor variations, our findings indicate that they are sufficient to significantly reduce antibody recognition of Chirp9 (Figure 5A,B). In contrast, the Chirp7 toxin possesses 18 distinct amino acid residues when compared to Chirp9. These differences include L5I, N7D, H8Y, S9H, E15T, F17A, L26V, Q31L, Q32R, G34Y, K35Q, G36S, G38H, G45A, Q54A, K62N, T64R, and N66K (Figure 5C), suggesting that both toxins might expose distinct sites to interact with antibodies.

**Figure 6:**
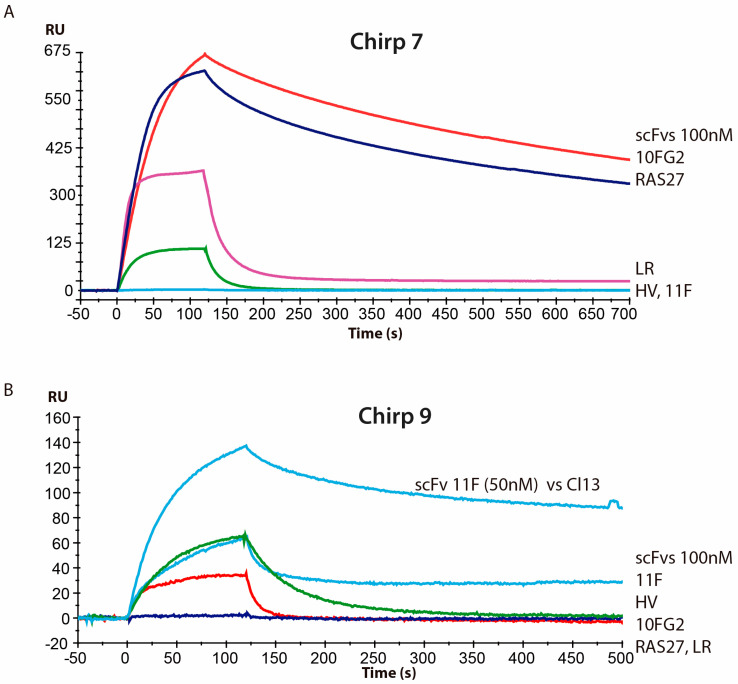
Sensorgram of interaction between scFvs and toxins. (**A**) with Chirp7 and (**B**) against Chirp9. Panel (**B**) shows the interaction of scFv 11F with toxin Cl13, which neutralizes it, contrasting with the low level of interaction with toxin Chirp9. Each scFv sensorgram is represented by a color. The *y*-axis represents Response Units (RU), the standard measure of binding signal in surface plasmon resonance (SPR), proportional to the amount of antibody bound to the sensor surface.

Antibody fragments LR, 10FG2, 11F, HV, and RAS-27, which were previously generated against other toxins in Mexican scorpion venoms, were tested for their ability to recognize the new toxins identified in the venom of *C. hirsutipalpus* [28]. Following a specified protocol, these antibodies, in single-chain variable fragment (scFv) format, were tested at a concentration of 100 nM. Sensorgrams obtained from each injection were compared (Figure 6). For the Chirp7 toxin, both the 10FG2 and RAS27 antibodies demonstrated strong recognition, as expected. The sensorgrams showed a significant level of interaction, characterized by robust association during the initial 120 s and minimal dissociation over the entire 700 s evaluation period. Previous studies on Ct1a toxin determined affinity constants (*K_D_s*) of 8 nM for scFv 10FG2 and 0.78 nM for scFv RAS27 [28]. These values suggest that both antibodies have the potential to neutralize the toxin. In contrast, the Chirp9 toxin exhibited low recognition by all evaluated antibodies. Notably, only scFv 11F, which was initially obtained through directed evolution against the Cl13 toxin, did not fully dissociate from Chirp9; however, its association level is still low. This suggests that the structural differences between the Chirp9 toxin and the Cl13 toxin might impact the interaction with scFv 11F, leading to reduced binding.

Biacore recognition analysis revealed that the most abundant venom component, toxin Chirp7, is recognized and potentially neutralized by antibodies 10FG2 and RAS27 [28], showing higher binding intensity and slow dissociation rates. Given its abundance and low level of neutralization, it will be necessary to further mature the scFv 11F antibody against toxin Chirp9 to achieve effective neutralization of this second component and, consequently, of the whole venom.

## 3. Conclusions

This study enabled the identification and characterization of two beta-type sodium toxins from the venom of *Centruroides hirsutipalpus*. These toxins, Chirp7 and Chirp9, exhibited modulatory activity on voltage-gated sodium channels and demonstrated lethality in mammals, confirming their biomedical relevance. Structural and functional analyses, including mass spectrometry, Edman degradation sequencing, electrophysiological studies, and antibody interaction assays, provided robust evidence of their role in scorpion envenomation and reinforced the value of these peptides as tools for ion channel research and as potential targets for the development of more specific antivenoms.

## 4. Materials and Methods

### 4.1. Biological Material

The scorpion specimens were collected in June 2017 from Minatitlán, Colima, Mexico. The collection coordinates are latitude 19°23′01.73″ N; longitude 104°03′35.19″ W; elevation is 703 m above sea level. The collection process was conducted with an official SEMARNAT permit (SGPA/DGVS/12063/15). Venom extraction from scorpions was performed by applying a 15-volt electrical stimulation on the telson. To estimate the average venom yield per scorpion, specimens were organized into groups of 50 individuals, except for one group of 20, resulting in a total of 220 collected scorpions. Each group was milked separately, and the venom from each set was pooled into a single tube, generating four tubes containing venom from 50 scorpions each and one tube containing venom from 20 scorpions. The collected venom was dissolved in water and clarified by centrifugation at 14,000 rpm for 15 min. The soluble fraction was recovered, and its absorbance at 280 nm was measured, assuming that one absorbance unit corresponds to 1 mg/mL of venom. The venom quantity for each group was calculated in micrograms and normalized to the number of scorpions in that group to determine the mean venom yield per individual and its standard deviation. The clarified venom was lyophilized and stored at −80 °C, conditions known to preserve scorpion venom stability and biological activity over extended periods.

### 4.2. Venom Fractionation and Purification of Toxins

The venom was fractionated using molecular exclusion chromatography. The soluble venom was passed through a Sephadex G-50 column (0.9 × 200 cm) equilibrated with 20 mM ammonium acetate at pH 4.7 and with a flow rate of 50 mL/h. Fractions of 5 mL were collected and quantified by absorbance at 280 nm. These fractions were subjected to ion-exchange chromatography using a Fast Protein Liquid Chromatography (FPLC) NGC^TM^ Chromatography System (Bio-Rad; Hercules, CA, USA) with a carboxymethylcellulose column (1 × 5 cm) equilibrated with 20 mM ammonium acetate at pH 4.7. The components were purified using a linear solvent gradient of ammonium acetate ranging from 0.02 M to 0.5 M over a 500 min run, with a flow rate of 1 mL/min at 9 psi. Fractions of 3 mL were collected and separated using a wavelength of 280 nm. Toxicity assays were performed on purified fractions from ion-exchange chromatography (see methodology below: “Biological assays”), and lethal fractions were further separated by high-performance liquid chromatography (HPLC) on an analytical C18 reverse-phase column (4.6 × 250 mm) from Grace Vydac (Hisperia, CA, USA). Lyophilized fractions (0.75 mg protein) were dissolved in 500 µL of solvent A (0.12% trifluoroacetic acid in water) and applied to the column. Elution was performed with a linear gradient of solvent B (0.10% TFA (Trifluoroacetic Acid) in acetonitrile) up to 60% over 60 min at a 1 mL/min flow rate for Chirp7. For Chirp9, the gradient was from 0 to 40% of B over 60 min. The content of protein was estimated at 280 nm, and fractions were collected using a wavelength of 230 nm and then dried in a Savant Speed Vac SC210A apparatus (Thermo Fisher Scientific, Albertville, MN, USA). The fractions were purified again to isolate the peptides in their pure form. Another portion was subjected to mass analysis, while a separate portion underwent toxicological testing, and the remainder was utilized for electrophysiological analysis. For mass determination, the samples were analyzed using a LCQ Fleet mass spectrometer (Thermo Finnigan, San Jose, CA, USA).

### 4.3. Toxin Sequencing by Edman Degradation

The amino acid sequence was determined and shown to be the most representative peptide in toxicity and lethality tests. The toxins were reduced and carboxymethylated before undergoing Edman degradation [37]. The amino-terminal sequence was obtained by loading approximately one nmol of the sample onto a CD-Inmobilon membrane using a Shimadzu Protein Sequencer PPSQ-31A733A (Shimadzu Scientific Instruments, Columbia, MD, USA). Some peptides were digested with endoproteinase aspartic-N and others with V8. The digestion products were purified using reverse-phase HPLC, and the fragments were processed to obtain the entire sequence. The amino acid sequence was registered at UniProt (https://www.uniprot.org/, accessed on 6 June 2024). The theoretical mass was determined from the given sequences and the experimental molecular weight using the Expasy Protein Param tool (https://web.expasy.org/protparam/, accessed on 2 February 2024).

### 4.4. Biological Assays

Toxicity assays. Toxicity and lethality tests of FPLC and HPLC fractions containing the toxins were conducted on CD1 strain albino mice (*Mus musculus*) weighing 20 g. The mice received intraperitoneal injections of 30 µg of each fraction diluted in 100 µL of Phosphate-buffered saline (PBS), while control mice were injected with sterile water. The mice were monitored for 24 h. Symptoms were categorized as follows: Toxicity was classified as “non-toxic” when the mice exhibited normal physiological and behavioral patterns indistinguishable from the control group. A treatment was categorized as “toxic” when the animals developed signs of intoxication, such as excitability, excessive salivation, transient paralysis, breathing difficulty, diarrhea, impaired coordination or movement, or excessive tearing. These symptoms would appear after administration but fully resolve within 24 h without lasting effects. In contrast, a treatment was considered “lethal” when the mice showed severe manifestations of intoxication, including progressive paralysis, marked respiratory distress, convulsions, or complete loss of mobility, leading to death during the observation period as a direct consequence of exposure. The experiments were conducted in triplicate. The Bioethics Committee approved the Animal Welfare protocol used in this study (Project 413).

Recordings of macroscopic currents from sodium channels. Human voltage-gated sodium channels (hNav 1.1, hNav 1.2 and hNa 1.6) were stably expressed in HEK cells. Cells were maintained at 37 ◦C with 5% CO_2_ in a humidified atmosphere. The growth medium consisted of high-glucose Dulbecco’s Modified Eagle Medium (DMEM, Sigma Aldrich, Naucalpan de Juarez, Estado de Mexico, Mexico), supplemented with 10% Fetal Bovine Serum (ByProductos, Guadalajara, Jalisco, Mexico) and 500 μg/mL of the antibiotic G418 (Sigma Aldrich, Naucalpan de Juarez, Estado de Mexico, Mexico). The extracellular solution was composed of (in mM): 130 NaCl, 5 KCl, 2 CaCl_2_, 2 MgCl_2_, 10 HEPES, and 5 glucose, with the pH adjusted to 7.3 using NaOH. The intracellular solution contained (in mM): 105 CsF, 27 CsCl, 5 NaCl, 2 MgCl_2_, 10 EGTA, and 10 HEPES, with the pH also adjusted to 7.3 with CsOH. Solutions were delivered to the patched cell via an active perfusion system connected to a variable-speed syringe pump (Model A-99, Razel Scientific Instruments, Fairfax, VT, USA), with a perfusion rate of approximately 1 μL/s. Patch pipettes were made from borosilicate glass capillaries (Warner Instruments, Hamden, CT, USA) using a vertical puller (Model P-30, Sutter Instrument, Novato, CA, USA). The pipettes were filled with the internal solution and had resistance ranging from 1.5 and 3 MΩ. Sodium currents were elicited by 100 ms depolarization ranging from −110 to 30 mV in 10 mV steps, followed by a fully activating step at −10 mV for 50 ms. To better appreciate the effects of scorpion β-sodium toxins, channels were primed by means of a 5 ms depolarization at 50 mV, applied 50 ms before the depolarization steps, as previously described in Cestele et al. 1998 [17]. To ensure complete recovery from inactivation, cells were maintained at −120 mV for 280 ms before each step, while holding potential was set at −100 mV. Currents were acquired using a MultiClamp 700A amplifier and a DigiData 1440A digitizer (Molecular Devices, CA, USA).

For the activation curve, conductance (G) was calculated from current (I) elicited during depolarization steps by means of the equation G = I/(V_m_ − E_Na_), where I is the current elicited at membrane potential V_m_, and E_Na_ is the reversal potential of Na^+^ ions, calculated experimentally for each cell. Conductance was normalized to its maximal level and plotted against the corresponding membrane potential. For the steady-state inactivation curve, currents elicited at −10 mV (after preconditioning depolarization from −110 to 30 mV) were plotted against the preconditioning potentials. Data from activation and from steady-state inactivation were fitted using a Boltzmann equation: *y* = *A*_2_ + (*A*_1_ − *A*_2_)/(1 + *exp*((*x* − *x*0)/*dx*)) where A_1_ is the initial value, A_2_ is the final value, *x*0 is the potential of half-activation (or half-inactivation) and *dx* is the slope.

For hNav 1.6 currents, activation data after toxin exposure were fitted using the sum of two Boltzmann equations: *y = y*0 + *A* (*frac*/(1 + *exp*((*x* − *x*01)*/k*1)) *+* (1 *− frac*)/(1 *+ exp*((*x* − *x*02)/*k*2))) where the sum is 1, x01 and dx1 are the same as under control conditions, and “frac” represents the fraction of channels bound to the toxin. Data are presented as means ± standard error (SE). The paired sample *t*-test (also known as the dependent samples *t*-test) was applied by comparing, for each individual cell recorded, the electrophysiological parameter of interest (e.g., peak current, V½ of activation or inactivation) before toxin application and after toxin application. Significant difference was accepted with a confidence level “*p*” set at 0.05.

Prediction of three-dimensional structures. The complete toxin sequences were used as input for the structure prediction. Among various 3D protein structure prediction methods, we employed the I-TASSER (Iterative Threading ASSEmbly Refinement; https://zhanggroup.org/I-TASSER/, accessed on 14 June 2024; [38]) server. For the Cl13 toxin, the NMR structure (PDB ID: 6VXW) was utilized. The structures were visualized and represented using the PyMOL program (version 3.1.4.1).

Evaluation of the recognition capability of scFvs for the toxins of *C. hirsutipalpus*. Surface plasmon resonance (SPR) evaluations of the recognition capability of scFvs for the toxins of *C. hirsutipalpus*. The toxins from *C. hirsutipalpus* were used to evaluate the recognition capacity of antibody fragments previously generated against Mexican scorpion toxins in a biosensor that detects molecular interactions in real time (Biacore X100, Uppsala, Sweden) [39,40,41]. The toxins Chirp7 and Chirp9 were covalently immobilized onto the surface of CM5 Chips using the amino coupling kit, achieving binding levels of 200 RUs (1 RU corresponds to a change in surface concentration of approximately one pg of bound mass per mm^2^). Each pure toxin was solubilized in 10 mM 2-(N-morpholino) ethanesulfonic acid (pH 6) and bound to cell 2. Cell 1 in the sensor (without bound toxin) was used as a control. The scFvs LR, 10FG2, 11F, HV, and RAS 27 [21] were evaluated for their ability to recognize each toxin. The samples were solubilized in HBS-EP buffer (HEPES buffered saline, with EDTA (ethylenediaminetetraacetic acid) and P20 (surfactant, Tween 20) (Biacore) at a concentration of 100 nM, and 100 μL of the scFvs were injected over the chip at a flow rate of 50 μL/min at 25 °C, with a delay time of 500 s. The chip surfaces were regenerated with 10 mM HCl. The sensorgram shows the curve corresponding to the association and dissociation phases at a given concentration of each scFv. The resulting sensorgrams were evaluated using BIA-evaluation software version 3.1.

## Figures and Tables

**Table 1:** Mass and toxicity of the most representative peaks.

Fraction	RT (HPLC, min)	Mass (Da)	Toxicity
FII.1	34.12	7108.9	Slightly toxic
FII.2	28.33	7050	Non-toxic
FII.3	28.4	6604.09	-
FII.4	32.26	7155.58	-
FII.5	32–38.5 *	7591.4	Toxic
FII.6	32–38.5 *	7591.4	-
FII.7 (Chirp7)	35.8	7590	Lethal
FII.8	29.96	7559	-
FII.9 (Chirp9)	50.4	7790.1	Lethal

* RCMC: Retention chromatographic conditions.

## Data Availability

The amino acid sequence was registered at UniProt (https://www.uniprot.org/), under accession number: Chirp7 (UniProt C0HMC5) and Chirp9 (UniProt C0HMC6).

## Supplementary Materials

The following supporting information can be downloaded at: https://www.mdpi.com/article/10.3390/toxins17120584/s1, Table S1. Fitting results of activation and inactivation processes of hNav 1.1, 1.2 and 1.6 channels before and after application of Chirp7 and Chirp9 at 200 nM. Fitting parameters for activation and inactivation processes: control condition is in no-shadow rows, while toxin treatment is in light gray shadow rows. V0.5 (mV): membrane potential of middle activation or inactivation; A: fraction of channels bound to the toxin; n.c.: not calculated. Data indicates the mean ± standard error of 3–5 cells. An asterisk (*) means significantly different at the 0.05 level in a paired sample *t*-Test. Figure S1. Recordings of macroscopic currents from sodium channels protocol and current traces.
