# Supplementary material for: Beta Toxins Isolated from the Scorpion Centruroides hirsutipalpus (Scorpiones; Buthidae) Affect the Function of Sodium Channels of Mammals
Source: Toxins (Basel). 2025 Dec 6;17(12):584. doi: 10.3390/toxins17120584 (PMC12737490; doi:10.3390/toxins17120584)
Supplement: Supplementary file 1 [file toxins-17-00584-s001.zip › toxins-3981693-supplementary.pdf]

Supplementary Materials: Beta Toxins Isolated from the Scorpion Centruroides hirsutipalpus (Scorpiones; Buthidae) Affect the Function of Sodium Channels of Mammals

Laura L Valdez-Velazquez, Timoteo Olamendi-Portugal, Rita Restano-Cassulini, Lidia Riaño-Umbarila, Juana María Jiménez-Vargas, Fernando Zamudio, Hermenegildo Salazar-Monge, Baltazar Becerril and Lourival D. Possani

Table S1. Fitting results of activation and inactivation processes of hNav 1.1, 1.2 and 1.6 channels before and after application of Chirp7 and Chirp9 at 200 nM.

			**hNav 1.1**	**hNav 1.2**	**hNav1.6**
**Chirp7**	**activation**	$V_{0.5}$ (mV)	-24.8 ± 0.8	-21.1 ± 0.4	-22.8 ± 0.3
			-26.9 ± 0.6*	-22 ± 0.4	-52.5 ± 3.4*
		slope	7.7 ± 0.7	5.4± 0.3	5.6 ± 0.22
			6.9 ± 0.5	5.9 ± 0.3	9.4 ± 1.9*
		A	n.c.	n.c.	0.34 ± 0.04
	inactivaiton	$V_{0.5}$ (mV)	-59.9 ± 0.2	-56.1 ± 0.2	-55.8 ± 0.3
			-60.9 ± 0.2	-56.9 ± 0.2	-58 ± 0.4
		slope	6.7 ± 0.1	6 ± 0.2	6.6 ± 0.3
			6.4 ± 0.1	6.2 ± 0.2	7.2 ± 0.4
		I/I_{max}	0.98±0.02	1 ± 0.02	0.88 ± 0.03*
**Chirp9**	activation	$V_{0.5}$ (mV)	-22.8 ± 0.3	-26.5 ± 0.5	-22.5 ± 0.3
			-23.6 ± 0.2	-26.8 ± 0.5	-40.9 ± 0.2*
		slope	7.2 ± 0.3	7.6 ± 0.4	7.0 ± 0.4
			8.2 ± 0.2	10.8 ± 0.4*	15.5 ± 0.9*
		A	n.c.	n.c.	1 ± 0.1
	inactivaiton	$V_{0.5}$ (mV)	-59 ± 0.2	-55.5 ± 0.3	-65.0 ± 0.1
			-60.1 ± 0.2	-61.8 ± 0.4*	-67.1 ± 0.5
		slope	6.8 ± 0.2	5.6 ± 0.2	6.3 ± 0.1
			7.3 ± 0.2	7.6 ± 0.4*	6.9 ± 0.4
		I/I_{max}	0.94 ± 0.02*	0.85 ± 0.07	0.29 ± 0.03*

Fitting parameters for activation and inactivation processes: control condition is in no-shadow rows, while toxin treatment is in grey shadow rows. $V_{0.5}$ (mV): membrane potential of middle activation or inactivation; slope: the parameter that describes the steepness of the transition between the lower and upper plateaus of the curve, A: fraction of channels bound to the toxin; n.c.: not calculated. Data indicates the mean ± SE of 5 cells. Asterisk (*) indicates significant difference at the 0.05 level under Paired Sample T Test condition.

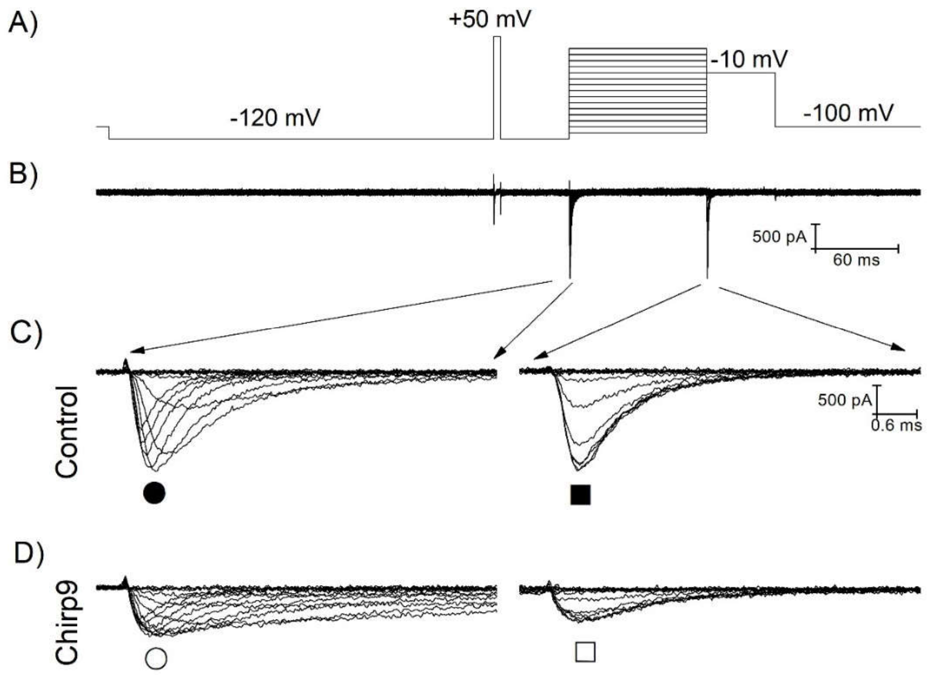

Figure S1. Recordings of macroscopic currents from sodium channels. Sodium currents in B) were elicited by the stimulation protocol depicted in A): currents were elicited by 100 ms depolarization ranging from -110 to 30 mV in 10 mV steps, followed by a fully activating step at -10 mV for 50 ms. A 5 ms depolarization at 50 mV was applied 50 ms before the depolarization steps to primed the channels. Peak currents obtained during the step depolarization from -110 to 30 mV (C and D, black circle in control and open circle after toxin application) were used to calculate the conductance and construct the activation curves. Currents recorded during the step at -10 mV (C and D, black square in control and open square for the toxin), were used to construct the inactivation curves.
